# Harnessing region-specific neurovascular signaling to promote germinal matrix vessel maturation and hemorrhage prevention

**DOI:** 10.1242/dmm.041228

**Published:** 2019-10-10

**Authors:** Devi Santhosh, Joe Sherman, Shafi Chowdhury, Zhen Huang

**Affiliations:** 1 Departments of Neuroscience and Neurology, University of Wisconsin-Madison, Madison, WI 53705, USA; 2 Program in Genetics and Medical Genetics, University of Wisconsin-Madison, Madison, WI 53705, USA

**Keywords:** Germinal matrix hemorrhage, Mouse model, TGFβ signaling, Brain neurovascular biology

## Abstract

Germinal matrix hemorrhage (GMH), affecting about 1 in 300 births, is a major perinatal disease with lifelong neurological consequences. Yet despite advances in neonatal medicine, there is no effective intervention. GMH is characterized by localized bleeding in the germinal matrix (GM), due to inherent vessel fragility unique to this developing brain region. Studies have shown that reduced TGFβ signaling contributes to this vascular immaturity. We have previously shown that a region-specific G-protein-coupled receptor pathway in GM neural progenitor cells regulates integrin β8, a limiting activator of pro-TGFβ. In this study, we use mice to test whether this regional pathway can be harnessed for GMH intervention. We first examined the endogenous dynamics of this pathway and found that it displays specific patterns of activation. We then investigated the functional effects of altering these dynamics by chemogenetics and found that there is a narrow developmental window during which this pathway is amenable to manipulation. Although high-level activity in this time window interferes with vessel growth, moderate enhancement promotes vessel maturation without compromising growth. Furthermore, we found that enhancing the activity of this pathway in a mouse model rescues all GMH phenotypes. Altogether, these results demonstrate that enhancing neurovascular signaling through pharmacological targeting of this pathway may be a viable approach for tissue-specific GMH intervention. They also demonstrate that timing and level are likely two major factors crucial for success. These findings thus provide critical new insights into both brain neurovascular biology and the intervention of GMH.

## Abstract (precis)

**Summary:** Temporal and dose-specific enhancement of regional neurovascular signaling improves germinal matrix vessel development and demonstrates, in principle, a new approach for germinal matrix hemorrhage intervention.

## INTRODUCTION

Neurovascular signaling is essential for normal blood vessel development in the brain. It underlies the development of the many unique properties of brain vessels that distinguish them from those of the rest of the body (Ma and Huang, 2015; Paredes et al., 2018). Endothelial cells (ECs) in the developing brain intimately interact with neural progenitor cells (NPCs), neurons and glia throughout different stages of development. Specific signals from neural cells coordinate the processes of EC proliferation, vessel ingression, stabilization and the formation of the blood-brain-barrier (BBB). More and more evidence has also linked vascular abnormalities to brain diseases (Li et al., 2018; Obermeier et al., 2013; Zhao et al., 2015), including potential roles played by defective neurovascular signaling. Thus, neurovascular interactions play critical roles in both normal brain development and in disease.

Although neurovascular signaling takes place across all different brain regions, increasing evidence indicates the existence of regional specificity. This is especially evident in neurological diseases such as germinal matrix hemorrhage (GMH). The germinal matrix (GM), a perinatal precursor to the postnatal striatum, has an increased incidence of neonatal intraventricular hemorrhage, a serious bleeding that occurs in ∼45% of low birth weight premature infants (Kadri et al., 2006; Lin et al., 2016). Studies have shown that GMH likely develops as a result of an intrinsic fragility of the vessels in this brain region. This in turn is likely a result of reduced basal lamina support components, an effect linked to a comparatively low level of TGFβ in the region (Ballabh, 2014), as well as a paucity of pericytes, a type of vascular support cell. This unique regional susceptibility of GM to hemorrhage thus suggests that although TGFβ signaling is uniformly required for blood vessel development throughout the brain, there exists region-specific mechanisms that regulate its activity.

TGFβ signaling plays multiple key roles in vascular development, regulating both EC proliferation and vessel stabilization and differentiation (Goumans et al., 2003). Whole embryo and EC-specific knockouts of TGFβ pathway components all lead to severe vascular problems and embryonic lethality (Jakobsson and van Meeteren, 2013). Despite this multitude of downstream roles, a common upstream regulator of latent TGFβ ligand activation in the brain is integrin αvβ8. Mutations in integrin αv (Bader et al., 1998; McCarty et al., 2002) and β8 (Proctor et al., 2005; Zhu et al., 2002) both result in compromised brain EC junction formation and hemorrhage. Intimate neuroepithelial-EC interactions have also been shown to play a crucial role in balancing the level and timing of active TGFβ for proper vascular development (Hirota et al., 2015). In addition, recruitment and maturation of pericytes is also regulated by TGFβ signaling (Li et al., 2011). Thus, TGFβ plays key roles in both EC and pericyte proliferation and differentiation during brain vessel development. These contrasting functions of TGFβ, regulating both vessel growth and maturation, highlight the importance of the regulation of the timing and level of TGFβ activity. Clearly, the dynamics of TGFβ signaling can strongly impact the downstream molecular and cellular responses in a context-dependent manner.

Previously, we have identified a region-specific pathway in GM NPCs that is vital for pro-TGFβ activation and vessel development in this region. We found that interference in NPC G-protein-coupled receptor (GPCR) signaling, through a *nestin-cre*-mediated deletion of a molecular chaperone (Ric8a) for Gα subunits throughout the brain, leads to vascular defects only in the GM (Ma et al., 2017). We further showed that these defects result from a reduction in integrin β8 gene (*Itgb8*) expression and consequently a decrease in active TGFβ. These results illustrate an indispensable role of NPC GPCR signaling in GM vessel development. More importantly, they identify a mechanism that is specific to the GM in the regulation of TGFβ signaling dynamics, at the level of transcription of the pro-TGFβ activator integrin β8 by this NPC GPCR pathway. This provides an opportunity not previously available to intervene in GMH and in a potentially tissue-specific manner.

To this end, we set out to harness the knowledge of this unique regional signaling pathway to prevent GMH. We first investigated the mechanisms underlying the unique pace of vessel development and maturation in the GM. We found that this unique pace is closely linked to the specific temporal dynamics of the GPCR signaling pathway. We then employed a chemogenetics approach to alter these GPCR signaling dynamics, to both determine their functional significance and to potentially use them to promote GM vessel maturation for GMH intervention. To achieve this, we expressed designer receptors exclusively activated by designer drugs (DREADDs) in GM NPCs (Armbruster et al., 2007). By administering the activating drug, clozapine-N-oxide (CNO), at different doses in a temporal-specific manner, we discovered a specific and narrow developmental window during which this pathway was amenable to manipulation. Although high doses of CNO inhibited GM vessel development, we found that a moderate dose improved GM vessel maturity without compromising growth. Furthermore, we found that CNO administration also rescued all GMH phenotypes in a mouse model that we generated. Thus, our study not only provides new mechanistic insights into the unique temporal regulation of GM vessel development but also demonstrates, in principle, a new and effective approach for GMH intervention and identifies the critical factors that underlie its success.

## RESULTS

### Temporal dynamics of NPC GPCR signaling in the GM

**Fig. 1.**
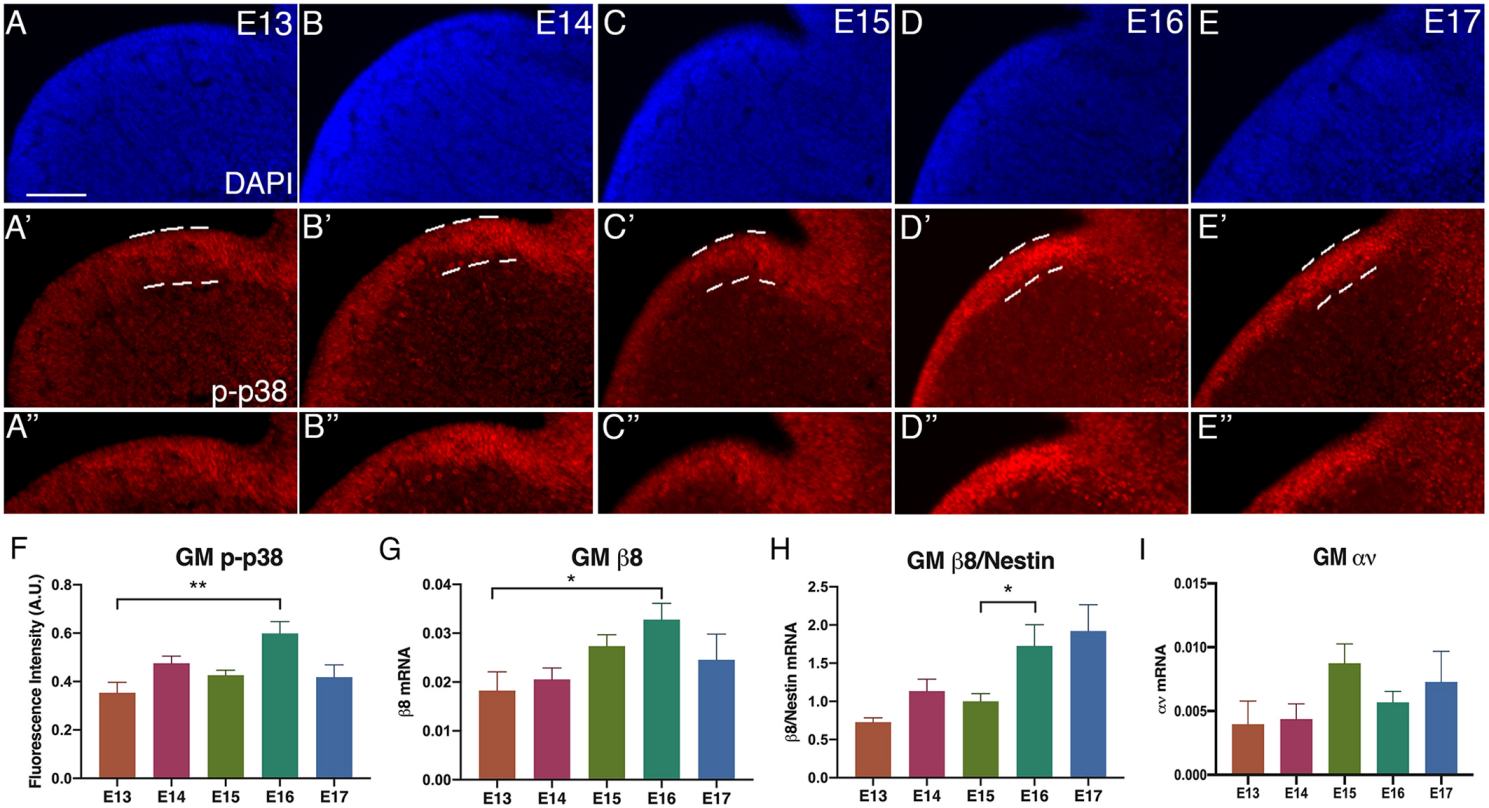
**Endogenous GPCR-integrin β8 signaling dynamics in the GM during development.** (A-E) DAPI images of GM from E13-E17. (A′-E′) Phospho-p38 (p-p38) staining of GM from E13-E17 in the ventricular zone (VZ, dashed lines). (A″-E″) Cropped images of phospho-p38 in the VZ. (F) Quantification of immunofluorescence of phospho-p38 staining (E13: *n*=9; E14: *n*=17; E15: *n*=18; E16: *n*=9; E17: *n*=11; **P=0.001). (G) q-PCR of integrin β8 gene expression from E13 to E17 in the GM (E13: *n*=5; E14: *n*=7; E15: *n*=6; E16: *n*=8; E17: *n*=5; *P=0.041). (H) Integrin β8 gene expression normalized to nestin gene expression for each embryonic stage (*P=0.020). (I) q-PCR of integrin αν gene expression from E13 to E17 (E13: *n*=8; E14: *n*=6; E15: *n*=6; E16: *n*=4; E17: *n*=6). Data are mean±s.e.m. ANOVA was used for statistical analyses. Scale bar: 100 µm.

We have previously shown that S1PR1 GPCR signaling in NPCs regulates the development of blood vessels in the GM (Ma et al., 2017). We showed that p38 (also known as Mapk14) is an essential mediator and integrin β8 is a transcriptional target of this pathway, which in turn regulates latent TGFβ activation. To establish the endogenous signaling dynamics, we used the activity of several components, including phosphorylated p38 (phospho-p38) and integrin β8 gene expression, as reporters for pathway activity. First, we measured the intensity of phospho-p38 in the ventricular zone of the GM from embryonic day (E)13 to E17 (Fig. 1A-E″). We found that phospho-p38 levels were moderate and relatively constant between E13 and E15, but then significantly increased at E16, by ∼69% compared with E13 (*P*=0.0011) and ∼40% compared with E15 (*P*=0.012) (fluorescent intensity, E13: 0.35±0.04; E15: 0.43±0.02; E16: 0.599±0.048; data are mean±s.e.m.), before declining at E17 (Fig. 1F). These results suggest a precise temporal regulation of this pathway in the GM.

A major downstream output of this pathway is integrin β8 mRNA expression. Thus, we measured integrin β8 mRNA as another indicator of this pathway activity (Fig. 1G). We found that the level of integrin β8 mRNA follows a trend gradually increasing from E13 to E16 (integrin β8 mRNA, E13: 0.18±0.003; E14: 0.2±0.002; E15: 0.027±0.002; E16: 0.03±0.003), with a significant increase of ∼79% by E16 in comparison with E13 (*P*=0.0411). As integrin β8 is primarily expressed in NPCs at these stages, we normalized the levels of integrin β8 mRNA against those of nestin mRNA (expressed almost exclusively in NPCs) to account for potential variability in tissue dissection. We found that, after normalization, integrin β8 mRNA shows relatively constant levels from E13 to E15, but then sharply increases at E16, by ∼137% in comparison with E13 (integrin β8/nestin mRNA, E13: 0.73±0.06; E14: 1.13±0.15; E15: 1.00±0.10; E16: 1.73±0.28; *P*=0.0204, ANOVA) (Fig. 1H). This pattern is very similar to that of phospho-p38 (Fig. 1F), which also shows an increase at E16. Thus, these results suggest that the increase in p38 activity at E16, likely from upstream GPCR activity, induces integrin β8 gene expression. Unlike the transient increase in p38 activity, however, the increase in integrin β8 mRNA level appears to persist at E17 (Fig. 1H). We surmise that this may be because of perdurance of integrin β8 mRNA. Thus, the activity and expression patterns of p38 and integrin β8 strongly suggest that there is a specific endogenous temporal dynamic of the GPCR pathway that regulates TGFβ activity and vessel development in the GM.

This specific pattern of activity of the GPCR-integrin β8 pathway may underlie the distinct pace of blood vessel development in the GM. To further assess their tissue specificity, we next investigated the temporal dynamics of p38 activity and integrin β8 expression in the developing neocortex. We found that, similar to that in the GM, phospho-p38 staining intensity in the cortical ventricular zone also showed an ∼2-fold increase from E15 to E16 (*P*=0.03) (Fig. S1A-F). However, the cortical phospho-p38 levels were consistently 2- to 5-fold higher than those in the GM across all stages from E13 to E17 (*P*<0.001 for all). As it is at present unknown whether p38 activity regulates the same pathway in cortical NPCs, this makes the significance of this dynamic unclear. To further explore this issue, we next directly analyzed integrin β8 mRNA levels (Fig. S1G). We found that, in contrast to those in the GM, integrin β8 mRNA levels in the cortex did not significantly change from E13 to E17 in a statistically significant manner (*P*>0.11 for all consecutive stage comparisons). Thus, these results indicate that GM and cortex show distinct patterns of p38 activation and integrin β8 induction. The particular dynamics of the GPCR-integrin β8 pathway in the GM are specific to this brain region and likely underlies its unique program of blood vessel development.

The GPCR-integrin β8 pathway regulates gene expression of integrin β8 but not that of its binding partner integrin αν (Ma et al., 2017), which also dimerizes with several other integrin β subunits. To further examine the specificity of the pathway dynamics, we also determined the expression of integrin αν (Fig. 1I). We found that, unlike that of integrin β8, integrin αν mRNA levels in the GM show an increase of ∼99% from E14 to E15 (E14: 0.004±0.001; E15: 0.0087±0.0015; *P*=0.024), one day earlier than integrin β8. As integrins αν and β8 are both essential to the formation of the ανβ8 dimer for latent TGFβ activation, this suggests that integrin β8 transcription is likely the limiting step in TGFβ activation and that its timing controls the temporal dynamics of GM vessel development. Thus, these results further indicate that the GPCR-integrin β8 pathway possesses specific temporal dynamics of activity in the GM. This strict temporal regulation also suggests a critical time window in GM vascular development, in which ECs may be transitioning from proliferating to differentiating.

### Chemogenetic manipulation of NPC GPCR pathway activity

**Fig. 2.**
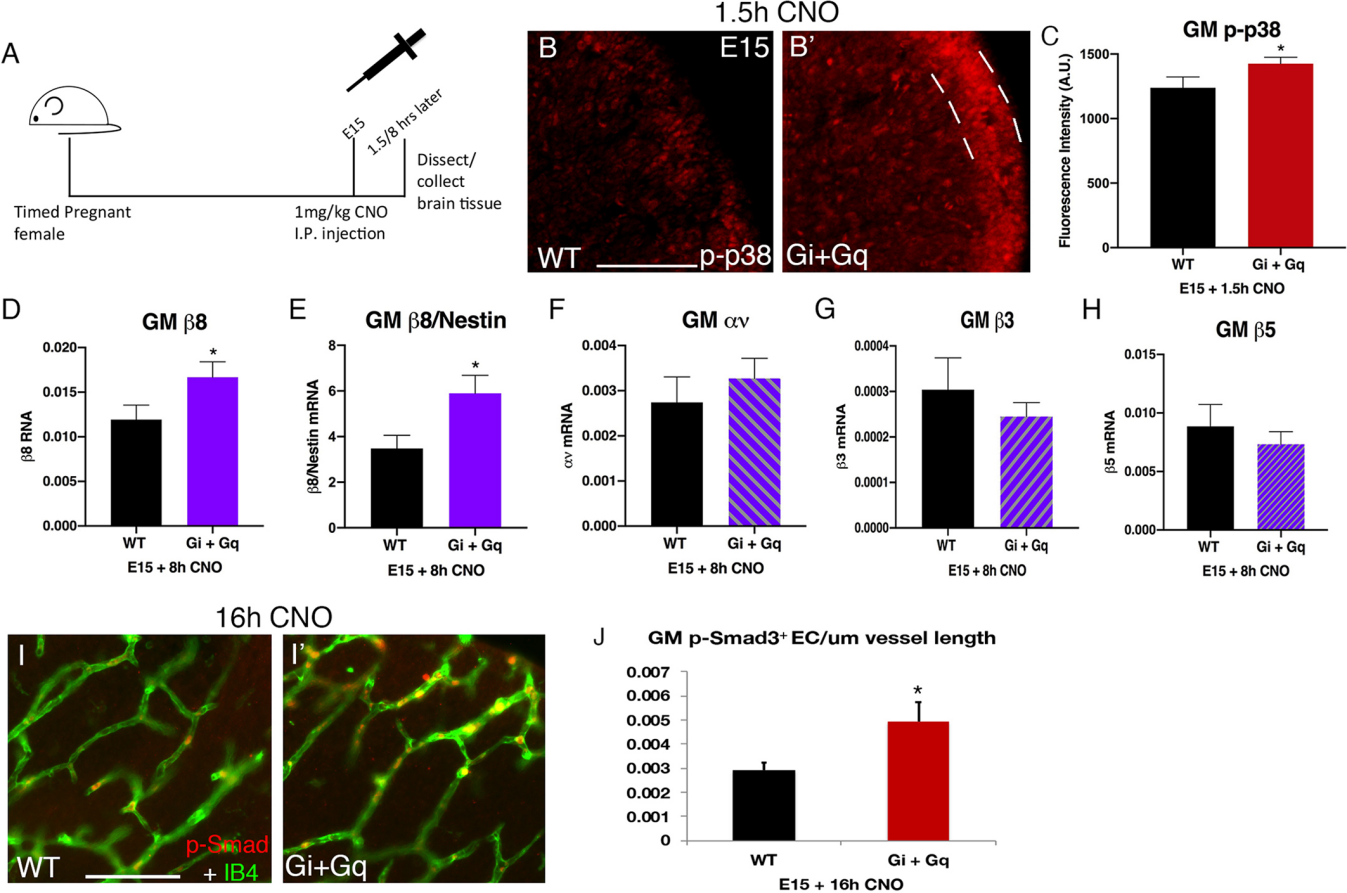
**DREADD activation at E15 specifically induces GPCR-integrin β8 pathway in the GM.** (A) Schematic of experiment showing IP injection of 1 mg/kg of CNO at E15 and tissue collection time points for analysis. (B,B′) Phospho-p38 (p-p38) staining in the ventricular zone (VZ) of the GM 1.5 h after CNO injection at E15 in wild-type (B) and Gi+Gq (B′) brains. Dashed lines indicate the VZ region quantified. (C) Quantification of phospho-p38 staining in B (WT+CNO: *n*=9; Gi+Gq+CNO: *n*=10; *P=0.03). (D) q-PCR of integrin β8 gene expression 8 h after CNO injection at E15 in wild-type and Gi+Gq brains (WT+CNO: *n*=12; Gi+Gq+CNO: *n*=8; *P=0.03). (E) q-PCR of integrin β8 gene expression normalized to nestin 8 h after CNO injection at E15 in wild-type and Gi+Gq brains (*P=0.01). (F-H) q-PCR of integrin αν (F), β3 (G) and β5 (H) gene expression 8 h after CNO injection at E15 in wild-type and Gi+Gq brains (WT+CNO and Gi+Gq+CNO: *n*=6; all *P*>0.1). (I,I′) Phospho-Smad3 staining along vessels in the GM 16 h after CNO injection at E15 in wild-type (I) and Gi+Gq (I′) brains. (J) Quantification of phospho-Smad3 staining in I (WT+CNO: *n*=9; Gi+Gq+CNO: *n*=11; *P*=0.016). Data are mean±s.e.m. One-way Welch's *t*-test was used for statistical analyses. Scale bars: 100 µm.

The tight regulation of p38 activity and integrin β8 mRNA levels strongly suggests that the temporal dynamics of the GPCR-integrin β8 pathway are fundamental in normal GM vessel development. To test this hypothesis, we took a chemogenetics approach. As Gαi (Gi-DREADD) and Gαq (Gq-DREADD) subunits are both known to associate with the S1PRs (Rosen et al., 2007) that regulate integrin β8 in GM NPCs (Ma et al., 2017), we employed two different DREADDs, one coupled to Gαi and the other to Gαq. We expressed these modified GPCRs in NPCs in a wild-type background using *nestin-cre* and the corresponding DREADD transgenic lines (Alexander et al., 2009). We injected 1 mg/kg body weight of CNO, the synthetic activating drug, into pregnant females to activate these receptors during embryonic development (Fig. 2A). We employed this dosage as it is the highest that has been shown to not have any non-specific effects on the brain (Gomez et al., 2017). To determine whether the GPCR-integrin β8 pathway is activated as predicted, we first measured the activity of p38 MAPK, a key pathway mediator that we have previously shown is both necessary and sufficient for inducing integrin β8 expression in GM NPCs. We found that 1.5 h after CNO injection at E15, active phospho-p38 staining was increased in the GM ventricular zone in brains expressing both Gi- and Gq-DREADDs (Gi/q) in comparison with non-expressing littermate brains (WT+CNO) (Fig. 2B,B′). Quantification showed that average phospho-p38 intensity increased by ∼15% in Gi/q-DREADD brains (WT+CNO: 1238.9±83.7; Gi/q-DREADD+CNO: 1425.6±49.8; *P*=0.03) (Fig. 2C). These results thus indicate that DREADD activation can lead to p38 activation in GM NPCs. To detect downstream effects, we next analyzed integrin β8 expression. We first performed analysis at 4 h after CNO injection and, although we observed a modest trend in increasing integrin β8 levels, it did not reach statistical significance (*P*=0.26, *n*=6). This suggests induction of integrin β8 mRNA may be a relatively slow process. We then analyzed gene expression 8 h after CNO injection at E15, and found that the integrin β8 mRNA level was significantly increased in Gi/q-DREADD-expressing brains, ∼by 40% compared with non-DREADD littermates (WT+CNO: 0.12±0.002; Gi/q-DREADD+CNO: 0.017±0.004; *P*=0.035) (Fig. 2D). To minimize tissue processing variability, we also normalized integrin β8 mRNA levels against those of nestin, which showed an ∼69% increase of integrin β8 (WT+CNO: 3.25±0.5; Gi/q-DREADD+CNO: 5.02±0.89; *P*=0.015) (Fig. 2E). Thus, these results further indicate that DREADD activation can activate the GPCR-integrin β8 pathway. Interestingly, at 24 h after CNO injection, we no longer observed significant differences in integrin β8 mRNA levels (*P*=0.44, *n*=5). This suggests that integrin β8 mRNA turnover is fairly quick, which is consistent with previous observations that integrin β8 transcription is a major factor in its regulation (Markovics et al., 2010). The pattern of a transient wave of integrin β8 induction immediately following CNO injection also supports the conclusion that this increase in expression is the result of DREADD activation. Furthermore, consistent with our observed tissue specificity of the GPCR-integrin β8 pathway, we did not observe a statistically significant difference in phospho-p38 (WT+CNO: 2010.24±62; Gi/q-DREADD+CNO: 1911.26±179; *P*=0.31) (Fig. S2A) or integrin β8 level in the cortex (WT+CNO: 0.03±0.008; Gi/q-DREADD+CNO: 0.055±0.007; *P*=0.08) (Fig. S2B). In addition, the gene expression of several other integrin subunits including αν (WT+CNO: 0.0027±0.0005; Gi/q+CNO: 0.003±0.001), β3 (WT+CNO: 0.0003±0.00007; Gi/q+CNO: 0.0002±0.00003) or β5 (WT+CNO: 0.008±0.002; Gi/q+CNO: 0.007±0.001) (all *P*>0.1) did not show statistically significant changes in the GM (Fig. 2F-H). Thus, these results together indicate that DREADD activation specifically induces a wave of integrin β8 expression in the GM.

Integrin αvβ8 promotes the cleavage and maturation of pro-TGFβ into active forms in the brain. To determine whether DREADD-induced integrin β8 results in predicted downstream effects, we next examined the activity of Smad2/3, an essential TGFβ pathway mediator, in GM ECs by analyzing Smad3 phosphorylation. We found that, in control non-DREADD littermates at 16 h after CNO treatment, there was a moderate number of phospho-Smad3-positive ECs along vessels in the GM (Fig. 2I). In contrast, the number of Smad3-positive ECs appeared substantially increased in DREADD-expressing animals 16 h after CNO injection (Fig. 2I′). Quantification showed that the density of phospho-Smad3-positive ECs was significantly increased, by >70%, in DREADD animals compared with their non-DREADD littermates (WT+CNO: 0.0029±0.0003/µm vessel; Gi/q+CNO: 0.0049±0.0008/µm vessel; *P*=0.016) (Fig. 2J). Thus, these results indicate DREADD-induced integrin β8 expression also results in the predicted increase in downstream TGFβ pathway activity.

### Stage-specific effects of DREADD activation on GM vessel development

**Fig. 3.**
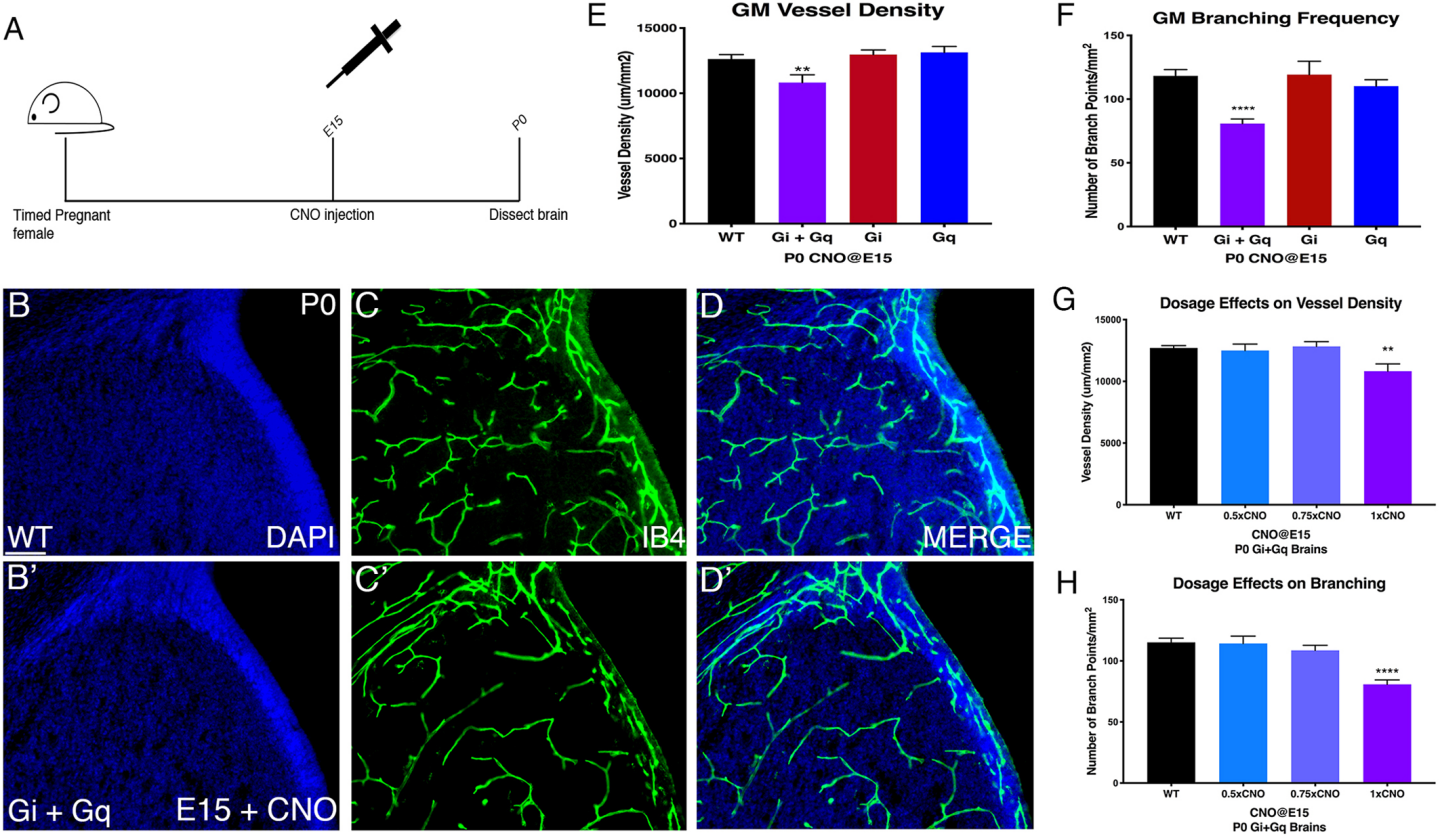
**GM vessel morphology is altered at P0 after E15 maximum CNO injection only in Gi+Gq brains.** (A) Schematic of experiment showing IP injection at E15 and analyzing vessel morphology at P0. (B,B′) DAPI staining of GM of wild-type (B) and Gi+Gq (B′) P0 brains after CNO injection at E15. (C,C′) IB4 staining of GM vessels in wild-type (C) and Gi+Gq (C′) P0 brains after CNO injection at E15. (D,D′) DAPI and IB4 merged images. (E,F) Quantification of vessel density (WT+CNO: *n*=21; Gi+Gq+CNO: *n*=13; Gi+CNO *n*=10; Gq+CNO: *n*=12; **P=0.009) (E) and branching frequency (WT+CNO: *n*=21; Gi+Gq+CNO: *n*=13; Gi+CNO *n*=10; Gq+CNO: *n*=12; ****P<0.0001) (F) in GM in wild-type, Gi+Gq, Gi alone and Gq alone brains after 1 mg/kg CNO injection at E15. (G,H) Quantification of vessel density (WT+CNO: *n*=53; 0.5×CNO: *n*=10; 0.75×CNO: *n*=17; 1×CNO: *n*=13; **P=0.0011) (G) and branching frequency (WT+CNO: *n*=39; 0.5×CNO: *n*=10; 0.75×CNO *n*=31; 1×CNO: *n*=13; ****P<0.0001) (H) in wild-type and Gi+Gq brains after a single injection of 0.5, 0.75 or 1 mg/kg of CNO at E15. Data are mean±s.e.m. ANOVA was used for statistical analyses. Scale bar: 100 µm.

The successful induction of integrin β8 by DREADDs provides an ideal tool to determine the functional significance of the unique temporal dynamics of the GPCR-integrin β8 pathway in GM vessel development. We have found that integrin β8 levels in the GM *in vivo* are normally low at E15 but become elevated after E16 (Fig. 1H). We thus scrutinized the biological significance of this transition by examining potential effects of premature elevation of integrin β8 levels at E15. To this end, we administered CNO as in Fig. 2, but analyzed vessel morphology at P0 (Fig. 3A). Interestingly, we found that CNO injection at E15 resulted in a reduction in vessel density and branching in the GM (Fig. 3B-D′). Quantification showed an ∼17% decrease in vessel density in Gi/q-DREADD-expressing brains compared with non-expressing littermates (WT+CNO: 12,623.92±341.4 μm/mm^2^; Gi/q-DREADD+CNO: 10,825.27±588.01 μm/mm^2^; *P*=0.009) (Fig. 3E). Vessel branching frequency also significantly decreased by ∼46% (WT+CNO: 118.24±5.01/mm^2^; DREADD+CNO: 80.8±3.59/mm^2^; *P*=0.0001) (Fig. 3F). In contrast, consistent with the lack of significant p38 activation or integrin β8 induction in the cortex (Fig. S2), DREADD activation at E15 did not alter cortical vessel density (WT+CNO: 11,807±528.8 μm/mm^2^; Gi/q-DREADD+CNO: 11,491±413.2 μm/mm^2^; *P*=0.65) or branching frequency (WT+CNO: 73.94±5.3 μm/mm^2^; Gi/q-DREADD+CNO: 73.45±4.9 μm/mm^2^; *P*=0.18) (Fig. S3A-E). To further evaluate that these effects are indeed due to CNO activation of DREADDs, we also analyzed untreated Gi/q-DREADD-expressing brains. We found that GM vessel density and branching were not affected in these brains (Fig. S3F,G). Thus, these results indicate that premature activation of the GPCR-integrin β8 pathway negatively affects normal vessel development in the GM.

To further analyze effects of DREADD activation, we next examined single Gi- or Gq-DREADD-expressing brains. Interestingly, we did not observe significant effects on either vessel density (Gi-DREADD+CNO: 12,968.29±351.48 μm/mm^2^; *P*=0.244; Gq-DREADD+CNO: 13,136.09±456.67 μm/mm^2^; *P*=0.19) or branching (Gi-DREADD+CNO: 119.2±10.6/mm^2^; *P*=0.47; Gq-DREADD+CNO: 110.2±5.1/mm^2^; *P*=0.13) in either genotype after E15 CNO injection (Fig. 3E,F). This suggests that single DREADD activation may not induce sufficient increases in integrin β8 expression. This is also in accordance with many previous studies showing synergistic interaction between Gi- and Gq-coupled GPCRs (Rebres et al., 2011). To determine potential dosage-dependent effects, we next injected several different doses of CNO (50% and 75% of the original full dose) at E15 and investigated the effects on Gi/q-DREADD-expressing brains at P0. We found that neither a 75% nor 50% dose affected either vessel density (Gi/q-DREADD+75% CNO: 12,829.12±385.2 μm/mm^2^; *P*=0.38; Gi/q-DREADD+50% CNO: 12,494.66±529.16 μm/mm^2^; *P*=0.35) or branching (Gi/q-DREADD+75% CNO: 108.2±5.9/mm^2^; *P*=0.11; Gi/q-DREADD+50% CNO: 114.28±5.9/mm^2^; *P*=0.45) (Fig. 3G,H). These results suggest that molecular pathways that normally regulate GM vessel development may be highly robust and that only strong interventions may lead to significant perturbation. Altogether, these results demonstrate that the temporally precise regulation of integrin β8 is critical to normal GM vessel development.

**Fig. 4.**
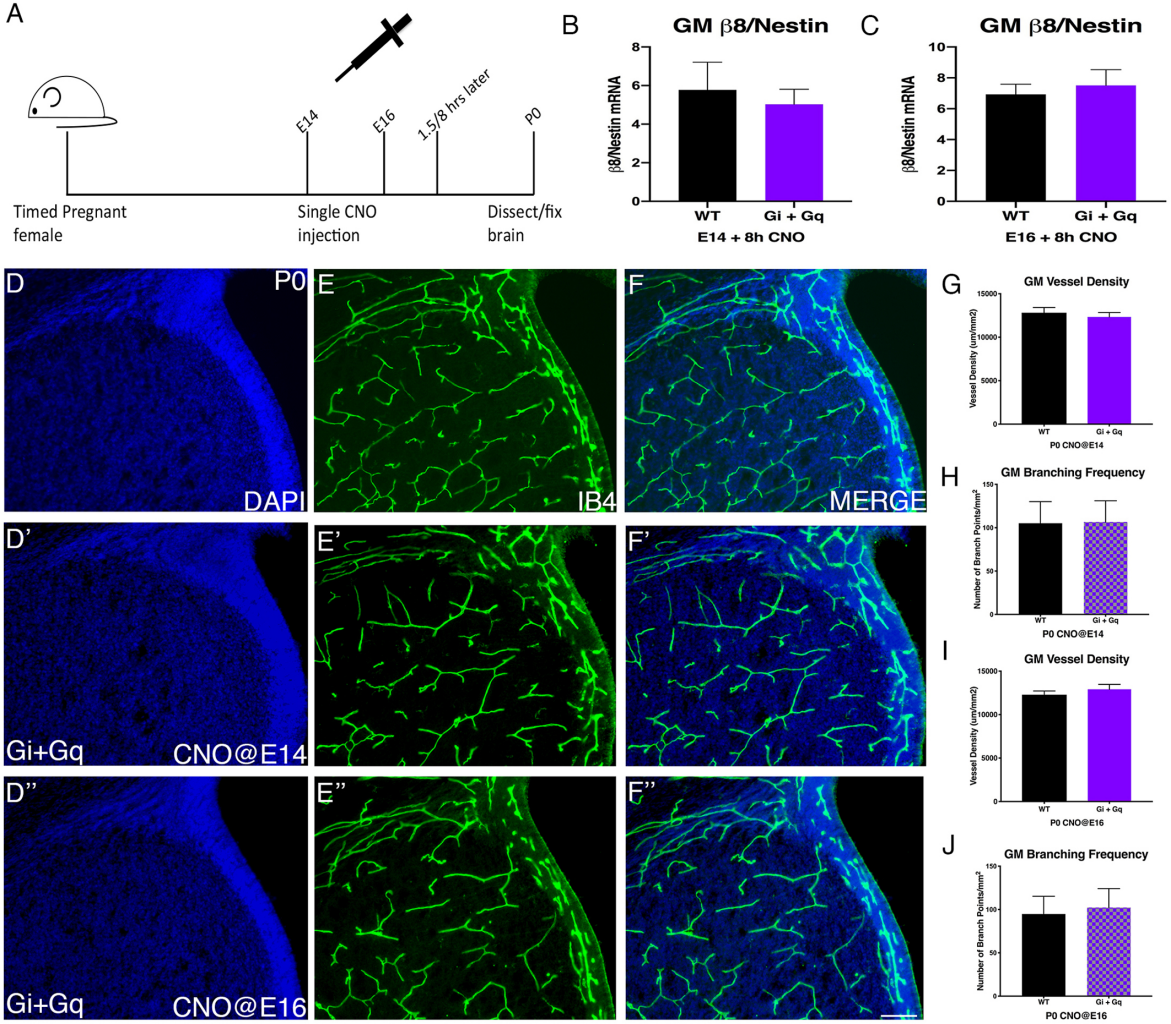
**Activation of DREADDs at E14 or E16 does not alter vessel morphology or integrin β8 expression in the GM.** (A) Schematic of experiment showing single IP injection of 1 mg/kg of CNO at either E14 or E16 and then the tissue collection time points for analysis. (B-C) q-PCR of integrin β8 gene expression 8 h after CNO injection at E14. (WT+CNO: *n*=6; Gi+Gq+CNO: *n*=6) (B) or at E16.5 (WT+CNO: *n*=5; Gi+Gq+CNO: *n*=5) (C). (D-D″) DAPI staining of GM of wild-type or Gi+Gq P0 brains after CNO injection at E14 (D′) or E16 (D″). (E-E″) IB4 staining of GM vessels in wild-type or Gi+Gq P0 brains after CNO injection at E14 (E′) or E16 (E″). (F-F″) Merge of DAPI and IB4 staining wild-type or Gi+Gq P0 brains after CNO injection at E14 (F′) or E16 (F″). (G-H) Quantification of GM vessel density (WT+CNO: *n*=10; Gi+Gq+CNO: *n*=16) (G) and branching frequency (WT+CNO: *n*=10; Gi+Gq+CNO: *n*=16) (H) after CNO injection at E14 in wild-type or Gi+Gq P0 brains. (I-J) Quantification of GM vessel density (WT+CNO: *n*=13; Gi+Gq+CNO: *n*=18) (I) and branching frequency (WT+CNO: *n*=13; Gi+Gq+CNO: *n*=18) (J) after CNO injection at E16 in wild-type or Gi+Gq P0 brains. Data are mean±s.e.m. One-way Welch's *t*-test was used for statistical analyses. Scale bar: 100 µm.

As premature elevation of integrin β8 at E15 negatively affects GM vessel development, we wondered whether similar results would occur at other stages. To test this, we next injected full-dose CNO at E14 and examined vessel morphology at P0 (Fig. 4A). Compared to wild-type brains (Fig. 4D-F), we found no significant changes in either vessel density (*P*=0.27) or branching (*P*=0.45) (Fig. 4D′-F′,G,H). To determine the cause of this lack of effects, we analyzed integrin β8 mRNA levels. We found that, unlike at E15 (Fig. 2D,E), integrin β8 mRNA levels showed no significant induction after CNO injection at E14 (*P*=0.41) (Fig. 4B). Similarly, we found CNO injection at E16 also did not cause any significant effects on either vessel density (*P*=0.19) or branching (*P*=0.37) (Fig. 4D″-F″,I-J). We also did not observe significant induction of integrin β8 mRNA at E16 (*P*=0.35) (Fig. 4C). Consistent with the lack of integrin β8 induction and unlike that following CNO injection at E15, we also did not observe significant changes in the density of phospho-Smad3-positive ECs after either E14 or E16 CNO injection (Fig. S4A,B). In addition, CNO injection at E11, E12, E13 and E17 also had no significant effects on vessel density or branching (Fig. S4C,D). Together, these results thus uncover a narrow sensitive window, around E15, during which the GPCR-integrin β8 pathway and neurovascular signaling in the GM appear to be susceptible to chemogenetic manipulation. They also suggest that, besides GPCR activation, there may exist additional mechanisms that contribute to the specific temporal dynamics of integrin β8 mRNA induction in the GM.

### Moderate-level DREADD activation promotes GM vessel maturation

**Fig. 5.**
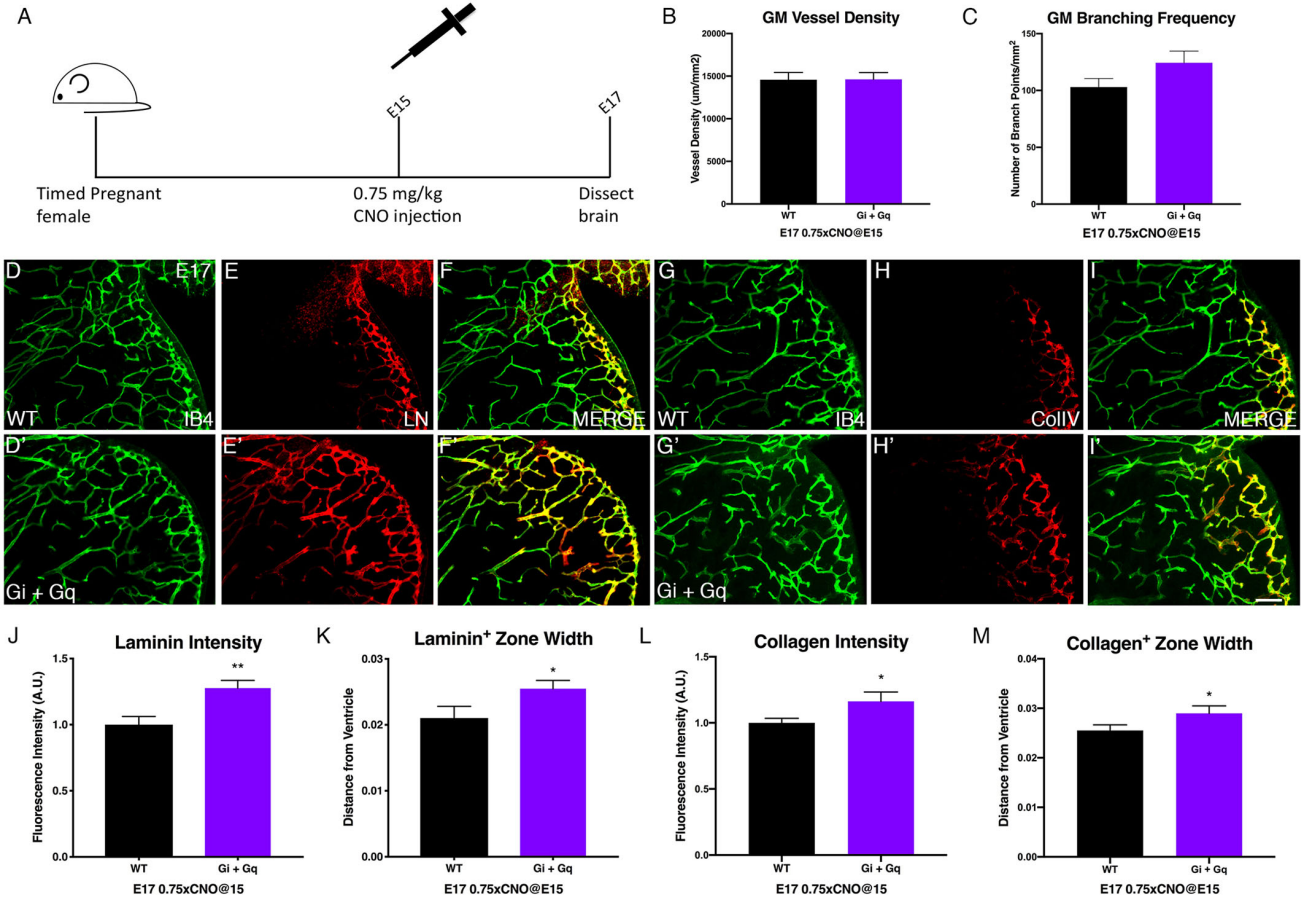
**Moderate dose of CNO injection at E15 enhances vessel maturity in the GM.** (A) Schematic of experiment showing single IP injection of 0.75 mg/kg of CNO at E15 and tissue collection at E17. (B,C) Quantification of vessel density (WT+CNO: *n*=8; Gi+Gq+CNO: *n*=11) (B) and branching frequency (WT+CNO: *n*=8; Gi+Gq+CNO: *n*=11) (C) in wild-type and Gi+Gq E17 brains after 0.75 mg/kg CNO injection at E15. (D,D′,G,G′) IB4 staining of GM vessels in wild-type (D,G) and Gi+Gq (D′,G′) E17 brains after CNO injection at E15. (E,E′) Laminin (LN) staining of GM in wild-type (E) and Gi+Gq (E′) E17 brains after CNO injection at E15. (F,F′) Merged image of IB4 and laminin from D,D′ and E,E′. (H,H′) Collagen IV staining of GM in wild-type (H) and Gi+Gq (H′) E17 brains after CNO injection at E15. (I,I′) Merged image of IB4 and collagen IV staining from G,G′ and H,H′. (J,K) Quantification of laminin fluorescence intensity (WT+CNO: *n*=13; Gi+Gq+CNO: *n*=11; **P=0.0036) (J) and staining positive zone (WT+CNO: *n*=13; Gi+Gq+CNO: *n*=14; *P=0.027) (K) in GM of wild-type and Gi+Gq E17 brains after CNO injection at E15. (L,M) Quantification of collagen IV fluorescence intensity (WT+CNO: *n*=17; Gi+Gq+CNO: *n*=17; *P=0.025) (L) and staining positive zone (WT+CNO: *n*=18; Gi+Gq+CNO: *n*=17; *P=0.038) (M) in GM of wild-type and Gi+Gq E17 brains after CNO injection at E15. Data are mean±s.e.m. One-way Welch's *t*-test was used for statistical analyses. Scale bar: 100 µm.

TGFβ signaling plays a critical role in promoting blood vessel maturation and integrity (Pardali et al., 2010). The human disease GMH is linked to reduced basement membrane components, such as laminin and collagen, and a paucity of pericytes in the GM (Braun et al., 2007; Xu et al., 2008). Both of these are regulated by TGFβ signaling. Thus, we wondered whether we could harness the GPCR-integrin β8 pathway to improve GM vessel maturation for the ultimate goal of intervening in GMH. To test this potential approach, we employed a 75% dose of CNO (0.75 mg/kg body weight) at E15 to activate DREADDs, as we have found that this dose does not adversely affect GM vessel morphology (Fig. 3G,H). We also analyzed GM vessels at E17 instead of at P0, as this stage, when the vessels are less mature than those at P0 (Fig. 5A), also correlates with the prenatal stage at which GMH typically occurs in premature human infants. As expected, we found that similar to results at P0 (Fig. 3G,H), 75% dose CNO injection did not significantly affect either vessel density (*P*=0.48) or branching (*P*=0.056) at E17 (Fig. 5B,C). To determine effects on GM vessel maturation, we next analyzed laminin and collagen coverage of GM vessels. Immunohistochemistry showed that both appeared to be enhanced at E17 in CNO-treated Gi/q-DREADD-expressing brains (Fig. 5D-I,D′-I′). Indeed, quantification showed that laminin intensity along vessels increased by ∼27% (*P*=0.0036) in Gi/q-DREADD-expressing brains compared with non-expressing littermates (Fig. 5J). The width of the laminin-positive zone near the lateral ventricle also increased by ∼21% (*P*=0.027) (Fig. 5K). Similarly, we found that collagen intensity along GM vessels also increased by ∼16% (*P*=0.025) and the width of the collagen IV-positive zone increased by ∼13% (*P*=0.038) (Fig. 5L,M). To determine the specificity of these effects, we also analyzed expression of Glut-1 (Slc2a1), a key BBB marker. We found that, unlike that of laminin and collagen, Glut-1 expression level was not significantly affected by CNO injection (*P*=0.94) (Fig. S5A,C). Furthermore, we found that the density of NG2-positive pericytes, needed for vessel stability, along GM vessels, although showing a moderate increasing trend, did not change in a statistically significant manner (Fig. S5D). Thus, these results indicate that moderate-level DREADD activation at the appropriate stage can promote GM vessel maturation, especially the deposition of basement membrane components, without adversely affecting overall vessel network development. This suggests that targeting the region-specific GPCR-integrin β8 pathway may be a feasible approach for GMH intervention.

### DREADD activation rescues GMH phenotypes in a disease model

**Fig. 6.**
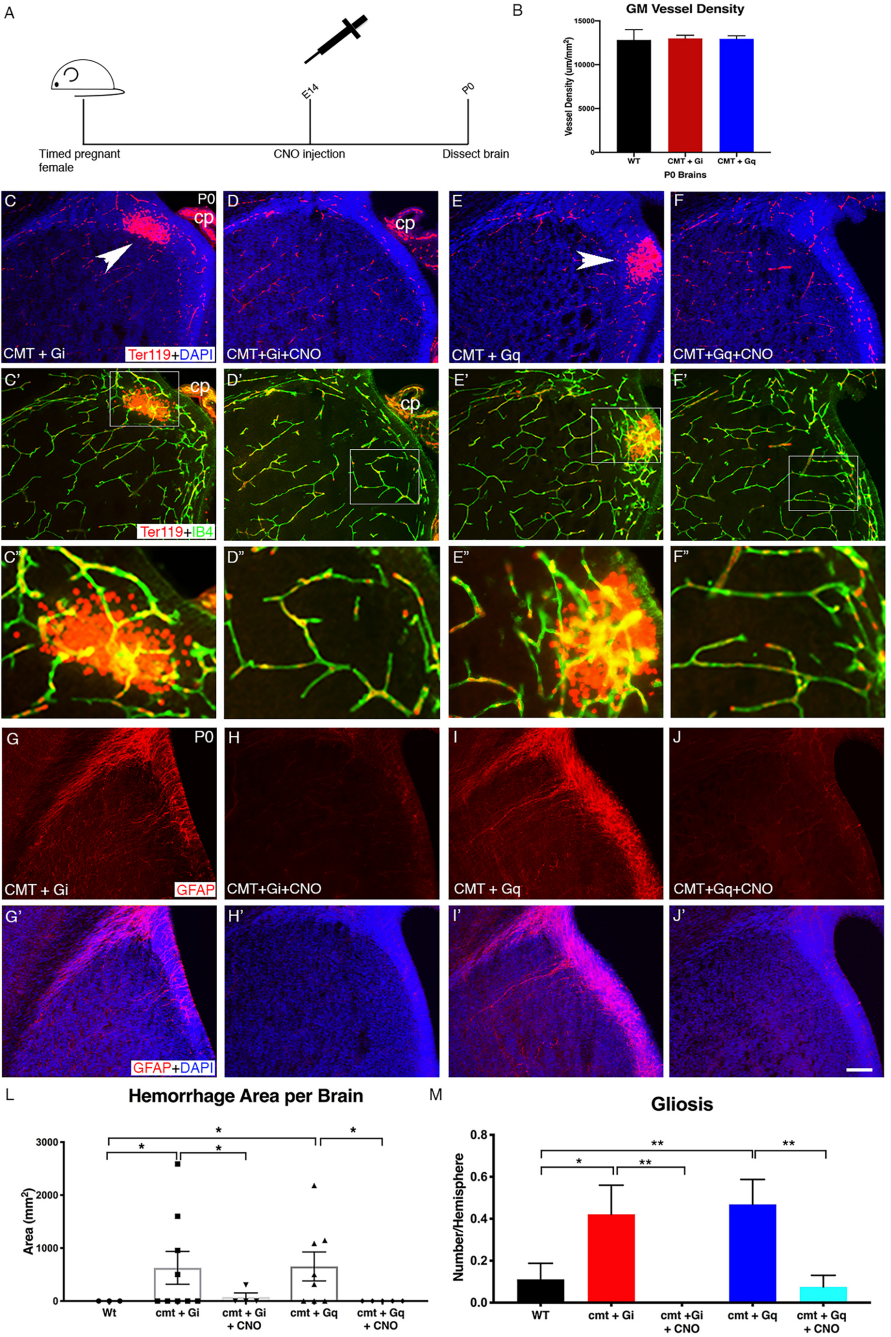
**DREADD activation rescues mouse model of GMH.** (A) Schematic of experiment showing CNO injection at E14 and analysis of brain tissue at P0. (B) Quantification of GM vessel density in wild-type, compound mutant CMT+Gi and CMT+Gq brains at P0 without CNO injection (WT: *n*=5; CMT+Gi: *n*=30; CMT+Gq: *n*=27). (C-F) Ter119 (red blood cell) and DAPI staining of GM in CMT+Gi (C) and CMT+Gq (E) P0 brains without CNO injection and GM after CNO injection in CMT+Gi (D) and CMT+Gq (F) P0 brains. Arrowheads indicate hemorrhage. (C′-F′) Ter119 and IB4 staining of GM in CMT+Gi (C′) and CMT+Gq (E′) P0 brains without CNO injection and GM after CNO injection in CMT+Gi (D′) and CMT+Gq (F′) P0 brains. (C″-F″) Magnification of boxed areas in C′-F′, respectively. Ter119-positive red blood cells can be clearly observed outside IB4-positive blood vessels in C″ and E″. (G-J) GFAP staining of GM in CMT+Gi (G) and CMT+Gq (I) P0 brains without CNO injection and GM after CNO injection in CMT+Gi (H) and CMT+Gq (J) P0 brains. (G′-J′) GFAP and DAPI staining of GM in CMT+Gi (G′) and CMT+Gq (I′) P0 brains without CNO injection and GM after CNO injection in CMT+Gi (H′) and CMT+Gq (J′) P0 brains. (L) Quantification of Ter119 staining in GM at P0 (CMT+Gi: *n*=9, *P*=0.038; CMT+Gq: *n*=8, *P*=0.024; CMT+Gi +CNO: *n*=4, *P*=0.038; CMT+Gq+CNO: *n*=5; *P*=0.024). (M) Quantification of GFAP staining in GM of P0 (CMT+Gi: *n*=19, *P*=0.03; CMT+Gq: *n*=32, *P*=0.007; CMT+Gi +CNO: *n*=15, *P*=0.0036; CMT+Gq+CNO: *n*=40, *P*=0.0022). Data are mean± s.e.m. cp, choroid plexus. One-way Welch's *t*-test was used for statistical analyses. *P<0.05, **P<0.005. Scale bar: 100 µm.

To more directly test the potential efficacy of activating the GPCR-integrin β8 pathway for GMH intervention, we next turned to a mouse model of GMH that we had previously generated (Ma et al., 2017). We demonstrated that homozygous deletion of Ric8a, a key heterotrimeric G protein co-factor, from NPCs results in GMH-like phenotypes including hemorrhage and gliosis specifically in the GM. We also showed that a compound mutant (cmt) of S1PR1 and Ric8a, in which both alleles of S1PR1 and one allele of Ric8a are deleted, resulted in similar GMH-like phenotypes. To determine the potential effectiveness of DREADD-mediated induction of integrin β8 in rescuing the GMH phenotypes, we employed the compound mutants, as in the Ric8a homozygous mutants all Go/i/q-dependent GPCR signaling would be completely blocked. We next introduced Gi- or Gq-DREADD into the compound mutant background (Fig. 6). We found that under this genetic background (and in the absence of CNO treatment), vessel morphology was relatively normal in both Gi- and Gq-DREADD-expressing compound mutants (Fig. 6B). However, severe hemorrhage (Fig. 6C-C″,E-E″) and gliosis (Fig. 6G-G′,I-I′) were observed in the brains of both mutants. Quantification of hemorrhage areas (WT: 0; cmt+Gi-DREADD, 627.4±339.12/mm^2^; *P*=0.038; cmt+Gq-DREADD: 654±273.4/mm^2^; *P*=0.024) and number of GFAP^+^ cells (WT: 0.111±0.076; cmt+Gi-DREADD: 0.4211±0.14; *P*=0.03; cmt+Gq-DREADD: 0.4688±0.12; *P*=0.007) confirmed the statistical significance of these observations (Fig. 6L-M). To determine effects of DREADD activation, we next injected CNO at E14 and examined P0 brains in these cmt DREADD mice (Fig. 6A). We selected E14 for CNO administration based on our previous results that either activation of p38 or ectopic expression of integrin β8 can rescue *Ric8a* mutant phenotypes at E14 (Ma et al., 2017). These experiments were all performed earlier and without knowledge of the temporal window we have described in wild-type animals. Importantly, we found that administering CNO at E14 effectively suppressed both hemorrhage (Fig. 6D-D″,F-F″) and gliosis (Fig. 6H-H′,J-J′) in both Gi- and Gq-DREADD-expressing mutant brains. Quantification confirmed that hemorrhaging was significantly reduced in both the cmt+Gi- or Gq-DREADD brains compared to those without CNO treatment (cmt+Gi with CNO versus without CNO: *P*=0.038; cmt+Gq with CNO versus without CNO: *P=*0.024) (Fig. 6L). Quantification of GFAP staining also showed that CNO injection led to a significant suppression of gliosis (cmt+Gi with CNO versus without CNO: *P*=0.0036; cmt+Gq with CNO versus without CNO: *P=*0.0022) (Fig. 6M). Thus, these results indicate that transient one-time exogenous activation of the GM-specific GPCR-integrin β8 pathway during development is sufficient to rescue vascular defects and associated damages in a GMH model. This provides strong proof-of-principle evidence that manipulation of this pathway during pregnancy may be a viable intervention for this disease in humans. Our findings that CNO administration at E14 is effective in a mutant but not wild-type background also suggest that different genetic backgrounds may shift or alter the time window of this GPCR-integrin β8 pathway sensitivity to manipulation and may be a factor that should be taken into consideration in clinical applications.

## DISCUSSION

**Fig. 7.**
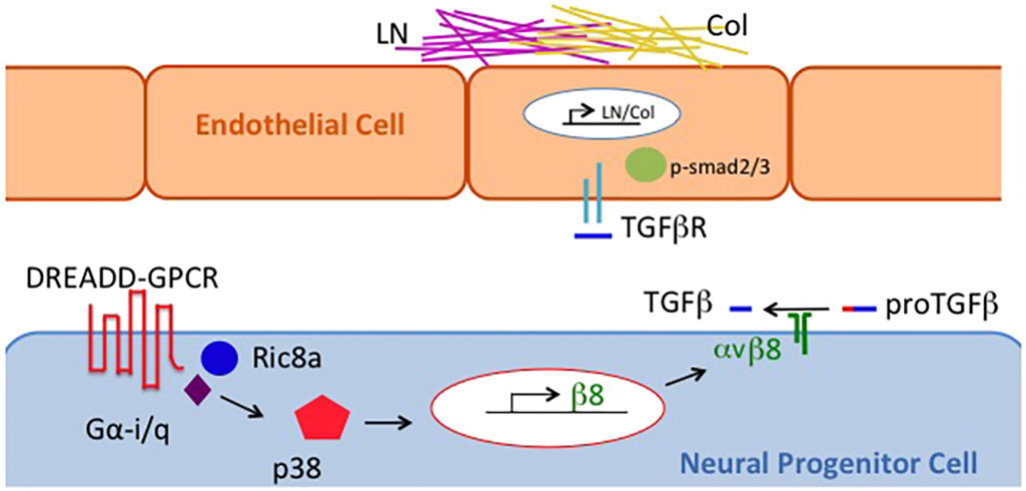
**Schematic diagram of DREADD-induced increases** **in basement membrane component deposition along blood vessels in the GM.** Ligand binding to DREADDs in neural progenitor cells results in Gαi/q and subsequently p38 activation, which then induces integrin β8 gene transcription followed by integrin αvβ8-dependent latent pro-TGFβ cleavage and activation. TGFβ activation of receptors on endothelial cells in turn signals through phosphorylated smad2/3 to increase expression of laminin (LN) and collagen (Col) which leads to increased deposition of the basement membrane components and, as a result, improved blood vessel integrity.

GMH is a major perinatal disease affecting about 1 in 300 births. Yet despite advances in neonatal medicine, there is currently no effective intervention. Studies have found that low TGFβ signaling underlies the inherent vessel fragility unique to this developing brain region that renders it susceptible to hemorrhage (Ballabh, 2014). TGFβ signaling is known to play complex roles in vessel development, regulating both vessel growth and maturation through dynamic fine-tuned activity (Goumans et al., 2003). We have previously identified a region-specific neurovascular pathway that regulates TGFβ signaling dynamics in the GM at the level of gene transcription of the upstream activator, integrin β8. In this study, we investigated the endogenous dynamics of this pathway *in vivo*, functionally tested their regulation, and successfully harnessed these dynamics for improving GM vessel maturation and intervening in GMH animal models (Fig. 7). We found that the activity of this pathway is finely tuned, with a distinct region-specific pattern that underpins the unique program of GM angiogenesis. We also found that the mechanisms that underlie this dynamic are highly robust and surprisingly resistant to exogenous perturbation. Importantly, we identified a specific developmental time window during which this pathway is amenable to modulation. We discovered that although high-level upregulation of this pathway during this time window disrupts normal vessel development, a refined level of modulation can significantly promote vessel maturation without affecting overall vessel development. Moreover, we found that activation of this pathway can also rescue GMH phenotypes in an animal model. Altogether, these results not only reveal novel insights into the mechanisms that underlie region-specific neurovascular regulation in the brain, but also provide strong proof-of principle evidence for a new tissue-specific approach for GMH intervention.

### Temporal regulation of the TGFβ-activating pathway in the GM

The GM has a specific program of blood vessel development unique to this brain region (Ma et al., 2017). Unlike other brain regions, it shows a slower pace of vessel maturation that has been linked to a low level of TGFβ signaling. We have previously shown that a key mechanism of TGFβ signaling regulation in the GM is an upstream region-specific GPCR-integrin β8 pathway that regulates the expression of integrin β8, a proteolytic activator of latent TGFβ. The identification of this pathway provides an opportunity to determine how the dynamics of TGFβ signaling in the GM underpins its unique program of angiogenesis. By measuring activity at several steps along this pathway, we discovered that there is a specific developmental switch from E15 to E16 when integrin β8 expression level increases in the GM. We also found that this temporal dynamic is distinct from that in the cortex. This suggests a critical region-specific temporal juncture in neurovascular signaling when activation of the GPCR-integrin β8 pathway kicks in a new stage of vessel development. Interestingly, studies of neurogenesis by NPCs in the GM have found that these integrin β8-expressing cells indeed undergo stage-specific transitions at these stages (Falk et al., 2017). This suggests coordination between neurogenesis and angiogenesis in the GM at many levels and further supports the interpretation of region-specific neurovascular regulatory biology throughout the brain.

To probe the functional significance of the observed signaling dynamics, we utilized a chemogenetics approach, using Gi- and Gq-coupled DREADDs (Armbruster et al., 2007). These synthetic GPCRs have been particularly useful for the study of neuronal circuits and behavior (Roth, 2016), though a few have also been employed in the study of non-neuronal cells (Guettier et al., 2009; Xie et al., 2017). They have, however, never before been used in the study of neurovascular development. To examine the consequences of altering the GPCR-integrin β8 pathway dynamics, we administered maximum specific CNO doses at various embryonic stages. Surprisingly, we found that at E15, and only at E15, was CNO administration able to induce integrin β8 and perturb vascular development. This has several folds of implications. First, as E15 is the stage immediately before the normal activation of the endogenous GPCR-integrin β8 pathway *in vivo*, this indicates that this precise temporal regulation is crucial for normal GM vessel development and premature activation will disrupt this process. Second, the existence of this small temporal window of susceptibility (E15 only) also suggests the existence of robust underlying mechanisms that regulate these endogenous temporal dynamics. Consistent with this observation, when we tested different doses of CNO, we found that only the highest non-off target dose (Gomez et al., 2017) disrupted vessel growth and branching. Furthermore, we observed these vascular effects only in brains expressing both Gi- and Gq-DREADDs, but not those expressing each alone. This further suggests that lower levels of pathway activation by each single DREADD may also be insufficient to overcome the endogenous regulation. It also suggests synergy between Gi- and Gq-DREADD pathways, which is in accordance with previous studies that showed synergistic crosstalk between Gi- and Gq-coupled GPCRs in numerous systems (Blaukat et al., 2000; Rebres et al., 2011). Altogether, these results reveal a previously unknown developmental switch in TGFβ activity regulation in the GM. They also suggest the existence of a robust underlying regulating system in which redundant mechanisms work together to ensure this precise switch. These results thus provide novel insights into the mechanisms that regulate neurovascular signaling and blood vessel development in the brain.

Our manipulation also affects only GM vessels, not vessels in neocortex, even though DREADDs are expressed in all NPCs throughout the brain. This is consistent with the regional specificity of the GPCR-integrin β8 pathway we described (Ma et al., 2017). These observations also highlight the question of regional differences in blood vessel development in the brain. It is now well accepted that neural and vascular functions are tightly coordinated throughout different brain regions. It is also well established that neural development in different brain regions is under compartmentalized regulation. Thus, different signaling mechanisms likely regulate the coordination of neurovascular development in different brain regions. Although integrin β8 expression is required for TGFβ activation and vessel development in both the GM and in the neocortex (Proctor et al., 2005; Zhu et al., 2002), we find that both endogenous and exogenous GPCRs only regulate integrin β8 in the GM but not in the cortex. This suggests different mechanisms must be in place in the cortex for integrin β8 regulation. Further investigations into these region-specific mechanisms may thus not only provide better insights into brain neurovascular development, but also uncover potential region-specific molecular targets for intervention into diseases that preferentially affect one brain region or another.

### Precise timing and dosing of TGFβ activity for GMH intervention

Despite advancements in treatment, GMH still has an incidence of 45% in extremely low birth weight infants (Wilson-Costello et al., 2005), which in turn can lead to severe neurological consequences ranging from developmental delays to post-hemorrhagic ventricular dilation, cerebral palsy and even death. The only current prevention against GMH in the clinic is to avert premature delivery using antenatal corticosteroids, but once hemorrhaging is detected, treatment strategies are limited to managing cranial blood pressure and minimizing abrupt changes in blood flow. In pre-clinical animal studies, a number of molecular targets have been identified and their potential for GMH intervention tested to different degrees (Ballabh et al., 2007; Yang et al., 2013). However, the efficacy of targeting molecules or pathways specific to the GM, which has the obvious advantage of potentially strongly reducing off-target effects, remains to be studied.

Low TGFβ signaling underlies the two factors linked to GMH: reduced vascular basement membrane components and pericyte coverage (Ballabh, 2014). Having established the tissue specificity of the upstream TGFβ-regulating GPCR-integrin β8 pathway and identified the specific time window when it is amenable to manipulation, we wondered whether we could leverage knowledge of this region-specific pathway to improve GM vessel development and intervene in GMH. To test the feasibility, we first determined whether altering TGFβ activity through DREADD-mediated modulation of integrin β8 can improve vascular maturity in the GM. To this end, we employed a dose that does not adversely affect overall GM vessel morphology, as identified through our dosage analysis. We found that inducing integrin β8 at this specific dosage was indeed sufficient to increase laminin and collagen coverage of GM vessels without inhibiting either vessel growth or branching. This enhancement of vessel maturity thus provides evidence that GM vasculature is amenable to improvement. To further test this strategy, we next employed a genetic model of GMH that we had previously generated. This model exhibits phenotypes, including hemorrhaging and gliosis, which closely resemble GMH in premature infants. We found that prenatal DREADD induction of integrin β8 also strongly ameliorates these defects, reducing both hemorrhaging and gliosis to wild-type levels. These results further demonstrate that targeting the tissue-specific GPCR-integrin β8 pathway can indeed be effective in GMH intervention and has significant therapeutic potential in humans. Although DREADD technology itself is obviously not feasible in humans, it is conceivable that small molecules may be discovered to specifically target this pathway through precise timing and dosage control for GMH intervention in humans. Moreover, we find that depending on the genetic background, the temporal window of pathway amenability to exogenous manipulation may also shift or alter in sensitivity. This indicates that patient-specific genetic background differences may need to be taken into consideration in clinical settings. Thus, altogether, our results not only provide strong proof of principle that validates the efficacy of a novel tissue-specific strategy in GMH intervention, but also identify critical factors including time and dosage that are key for success in its implementation. This lays a critical foundation for potential GMH intervention in the clinic through employing tissue- and pathway-specific pharmacological agents.

## MATERIALS AND METHODS

### Mice

To generate the DREADD mouse lines the TRE-hM3Dq [STOCK Tg(tetO-CHRM3*)1Blr/J], TRE-hM4Di [B6.Cg-Tg(tetO-CHRM4*)2Blr/J], and ROSA:LNL:tTA [B6.129P2(Cg)-Gt(ROSA)26Sor^tm1(tTA)Roos^/J] mice from The Jackson Laboratory were crossed. Along with S1PR1 and Ric8a conditional alleles (Ma et al., 2017), all lines were bred with *nestin-cre*, and maintained in a mixed 129/B6 background (for details see Table S1). Stage-matched animals (from embryos to neonates) were analyzed in blind regarding sex and littermates were used for comparative analysis whenever possible. Sample size was selected based on obtaining at least three independent animals of each genotype, including littermate controls for each experimental condition and analyzing more than one litter. Each experiment had at least three replicates for each condition, upon which statistical tests were performed to ensure minimal and consistent levels of variability, with the total number of replicates reported in the figure legends. Animal use was in accordance with institutional and national guidelines and regulations, and approved by the University of Wisconsin-Madison Institutional Animal Care and Use Committee.

### Immunohistochemistry

Mouse brains were sectioned at 40-50 µm using a vibratome (Leica Microsystems) and fixed in 4% paraformaldehyde overnight at 4°C. The following primary antibodies were used at respective dilutions/concentrations: rabbit anti-laminin (Sigma-Aldrich, 1:2000), rabbit anti-Collagen IV (Abcam, 1:400; Bio-Rad, 1:400), biotinylated Isolectin B4 (Vector Laboratories, 1:200), rat anti-Terr119 (BD Pharmingen, 1:200), rabbit anti-Phospho-p38 (Cell Signaling Technology, 1:400) and rabbit anti-GFAP (Dako, 1:500). FITC- and Cy3-conjugated secondary antibodies were purchased from Jackson ImmunoResearch Laboratories. Peroxidase-conjugated secondary antibodies were purchased from Santa Cruz Biotechnology. FITC-conjugated streptavidin was used as secondary for biotinylated isolectin B4. See Table S1 for details. Staining procedures were performed as previously described (Ma et al., 2013). Briefly, primary antibodies were incubated at 4°C, overnight, followed by three washes with normal serum, and secondary antibodies were incubated at room temperature for 4 h followed by DAPI (1:2000) for 20 min. For phospho-p38, we used a tyramide signal amplification (TSA) plus Cy3 kit (PerkinElmer, 1:1000) based on the manufacturer's instruction. After secondary antibody and DAPI staining, sections were mounted with Fluoromount G medium (SouthernBiotech) and analyzed using a Nikon eclipse Ti microscope.

### RT-qPCR

Cerebral cortices and lateral ganglion eminences were isolated from E13, E14, E15, E16 and E17 mouse embryos. Total RNAs were isolated using Trizol (Invitrogen) and cDNA prepared using a reverse transcription kit and random hexamer primers (Applied Biosystems). qPCR reactions were performed using the GoTaq qPCR master mix (Promega). mRNA levels were normalized to those of GAPDH and the ΔΔCT method used to determine expression level. See Table S1 for primer information.

### Pharmacology

For drug injection, pregnant females (staged based on plug date) were intraperitoneally (IP) injected with chemical compounds: clozapine-N-oxide {0.38 [∼0.5 mg/kg body weight (gbw)], 0.57 (∼0.75 mg/kg gbw), or 0.76 mM (∼1 mg/kg gbw)}. For the GMH rescue, mice were also injected for four days (E12-E15) with JTE-013 (0.6 µM) and TY52156 (3.3 µM) (see Table S1 for details of reagents and resources). Embryos or pups were collected for analyses as described in the Results section.

### Quantification and statistical analysis

Vessel morphology and immunofluorescence were quantified using Nikon NIS-Elements BR 3.0 and ImageJ software as previously published (Ma et al., 2013). Any data without technical errors was included, while outliers, though not defined before beginning of the study, were concurrently identified by robust statistical tests, excluded and not reported. Nonparametric Student's *t*-test with Welch's correction was used for data involving two conditions and one-way ANOVA followed by Tukey's post hoc test were employed for data involving more than two conditions. Any outliers were removed using the ROUT test. *P*<0.05 is considered statistically significant in all cases. Sample sizes and *P*-values are included in the figure legends. Error bars represent s.e.m.

## Supplementary Material

Supplementary information
