# Supplementary material for: Harnessing region-specific neurovascular signaling to promote germinal matrix vessel maturation and hemorrhage prevention
Source: Dis Model Mech. 2019 Oct 10;12(11):dmm041228. doi: 10.1242/dmm.041228 (PMC6899033; doi:10.1242/dmm.041228)
Supplement: Supplementary information [file dmm-12-041228-s1.pdf]

Supplemental Figures

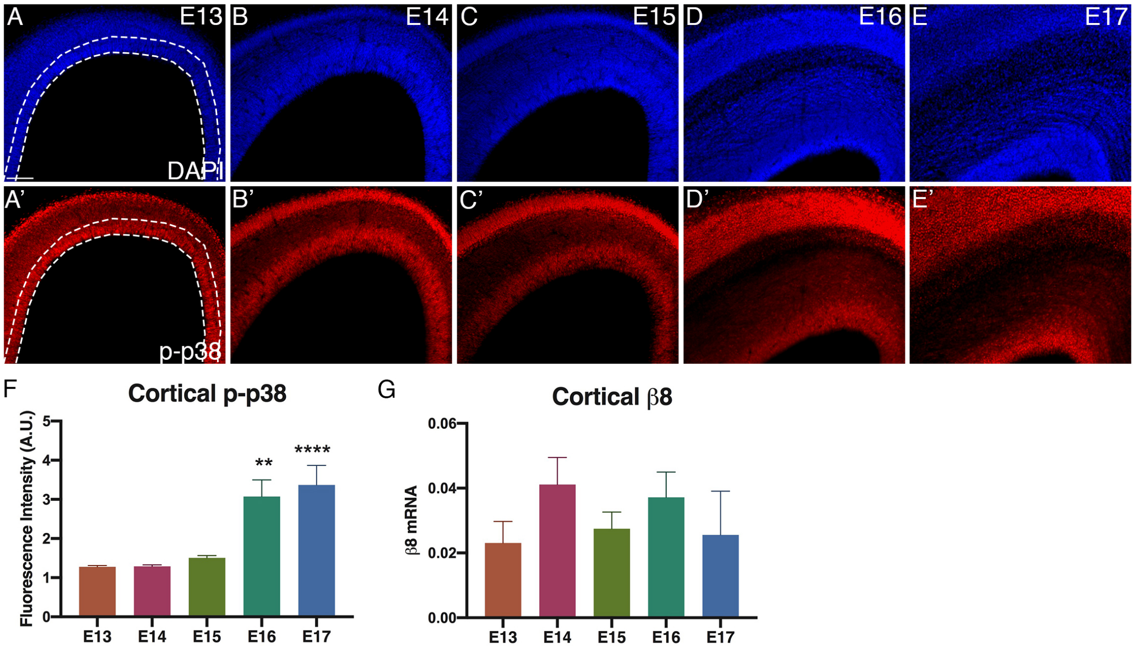

Figure S1: Endogenous cortical GPCR-β8 signaling dynamics during development

(**A-E**) DAPI images of cortex from embryonic day 13 (E13) to E17.

(**A'-E'**) Phospho-p38 (p-p-38) staining of cortex from E13-E17.

(**F**) Quantification of immunofluorescence of phospho-p38 staining. (E13: n = 5; E14: n = 10, E15: n = 12, E16: n = 3, E17: n = 7; $p = 0.0032, 0.0001$)

(**G**) Quantitative PCR (q-PCR) of integrin β8 gene expression from E13.5 E17.5 in the cortex. (E13: n = 8; E14: n = 7, E15: n = 5, E16: n = 7, E17: n = 4)

Disease Models & Mechanisms • Supplementary information

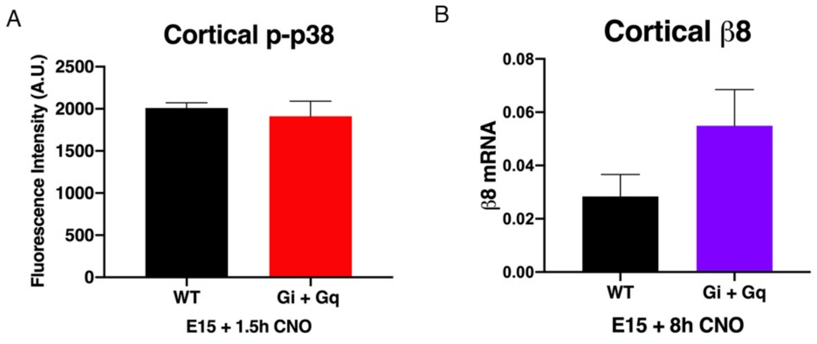

Figure S2: DREADD activation at E15 does not induce β8 pathway in the cortex

(**A**) Quantification of p-p38 cortical staining in WT and Gi+Gq brains 1.5 hours after CNO injection at E15 (ctl: n=9, Gi+Gq: n=7)

(**B**) q-PCR of integrin β8 gene expression 8 hours after CNO injection at E15 in wild type and Gi+Gq brains (ctl: n=5, Gi+Gq: n=4).

Disease Models & Mechanisms • Supplementary information

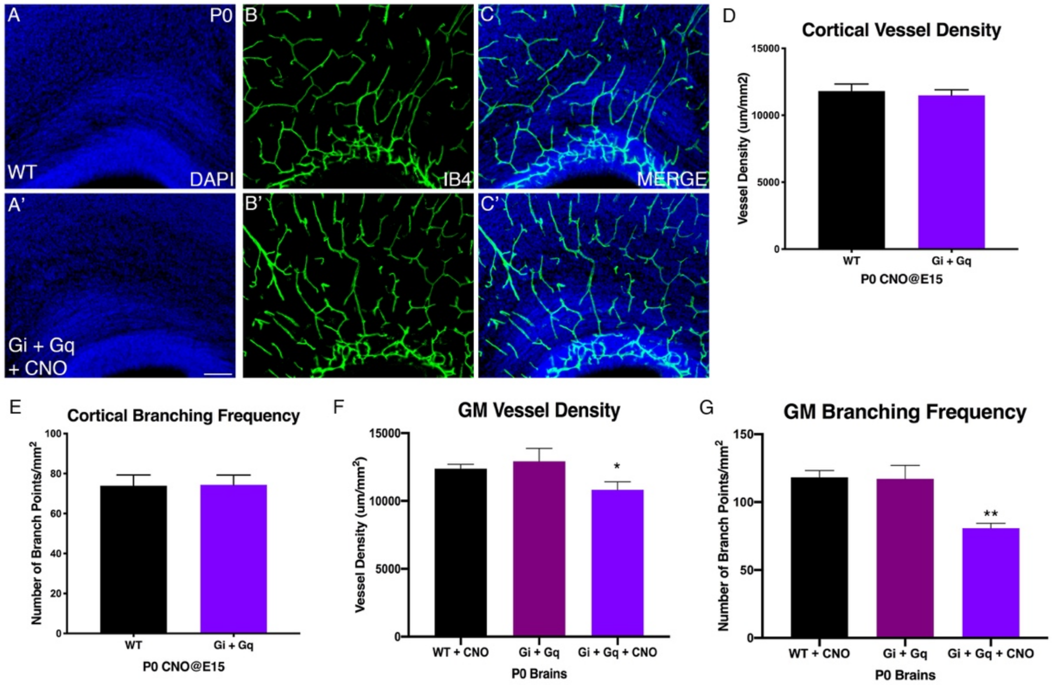

Figure S3: Cortical vessel morphology is not affected by E15 DREADD activation

(**A-A'**) DAPI staining of cortex of wild type and Gi+Gq P0 brains after CNO injection at E15.

(**B-B'**) IB4 staining of cortical vessels in wild type and Gi+Gq P0 brains after CNO injection at E15.

(**C-C'**) DAPI and IB4 merge

(D-E) Quantification of cortical vessel density (WT+CNO: n = 6, Gi+Gq+CNO: n = 7; $p = 0.65$) (D) and branching frequency (WT+CNO: n = 6, Gi+Gq+CNO: n = 7; $p = 0.32$) (E) in in wild type and Gi+Gq after 1 mg/kg CNO injection at E15.

(**F-G**) Quantification of GM vessel density in WT + CNO (n = 21), Gi+Gq without CNO (n = 14), and Gi+Gq + CNO (n = 13; $p = 0.002$) (F) and branching frequency WT + CNO (n = 21), Gi+Gq without CNO (n = 14), and Gi+Gq + CNO (n = 13; $p = 0.0004$) (G) in wild type and Gi+Gq brains after a single injection of 1 mg/kg of CNO at E15.

Disease Models & Mechanisms • Supplementary information

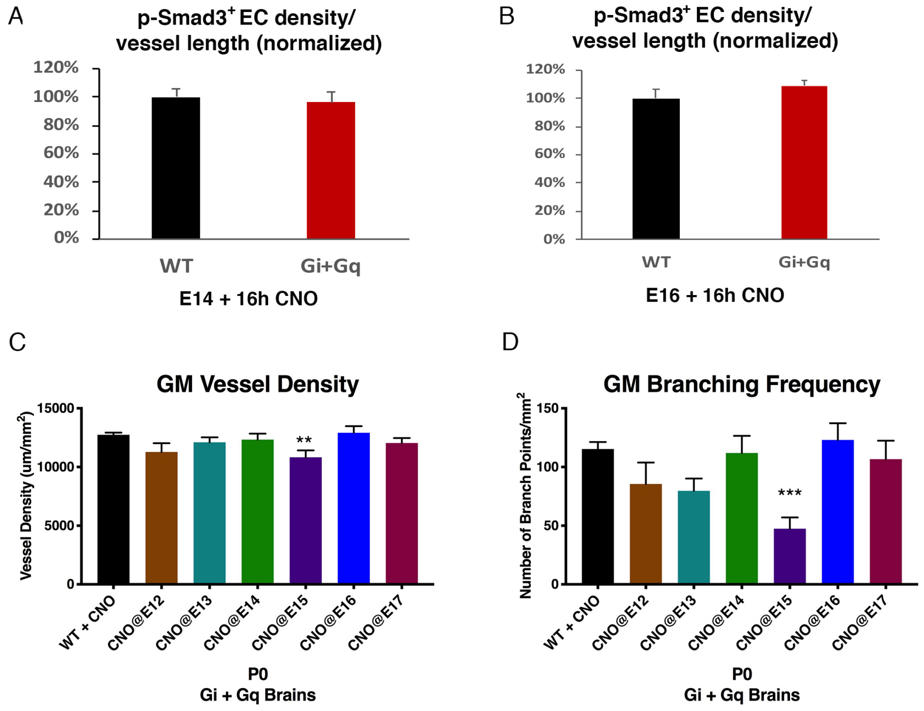

Figure S4: CNO injection at various developmental stages shows only E15 activation has effects on GM vessel morphology

(**A**) Quantification of phospho-Smad3-positive ECs along vessels in the GM 16 hours after CNO injection at E14 in wild type and Gi+Gq brains.

(**B**) Quantification of phospho-Smad3-positive ECs along vessels in the GM 16 hours after CNO injection at E16 in wild type and Gi+Gq brains.

(**C**) Quantification of vessel density in GM at P0 after single injection of CNO at one developmental stage in wild type and Gi+Gq expressing brains (E11-E17).

(**D**) Quantification of vessel branching in GM at P0 after single injection of CNO at one developmental stage in wild type and Gi+Gq expressing brains (E11-E17).

Disease Models & Mechanisms • Supplementary information

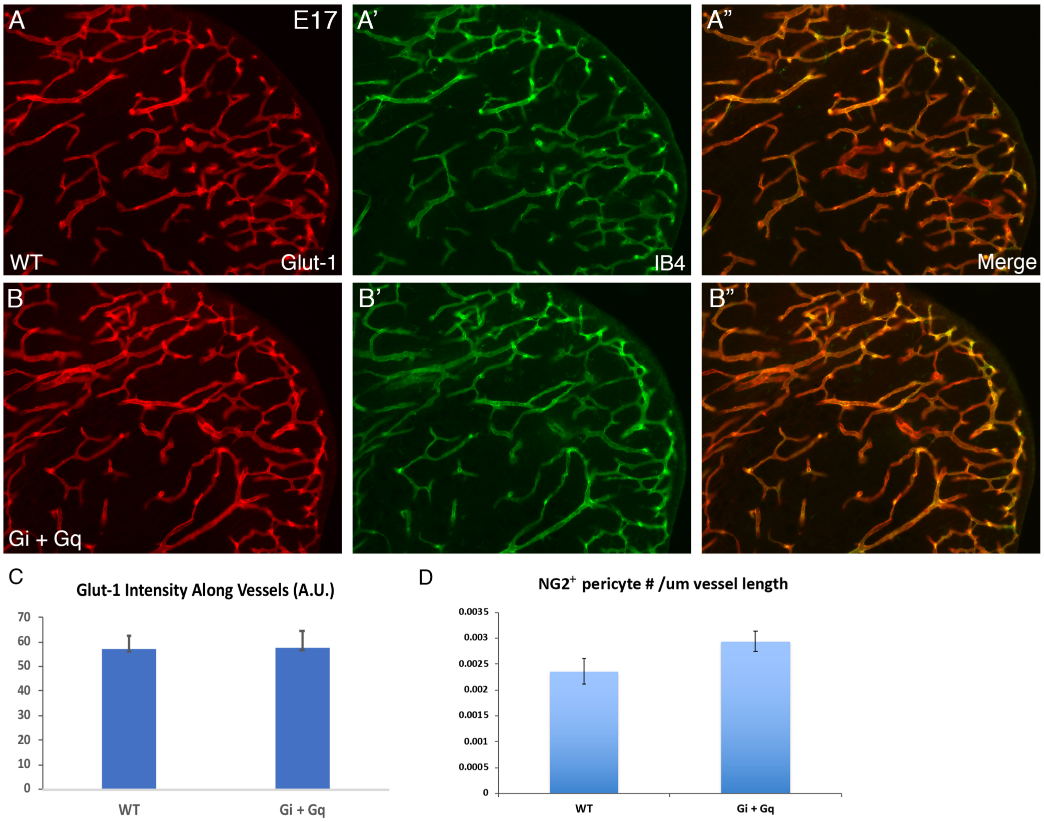

Figure S5. 0.75x CNO injection at E15 does not significantly affect Glut-1 expression or pericyte density.

(**A-B"**) Glut-1 and IB4 staining of GM vessels in wild type (A-A") and Gi+Gq (B-B") E17 brains after 0.75x CNO injection at E15.

(**C**) Quantification of Glut-1 intensity along vessels in the GM at E17 after 0.75x CNO injection at E15 in wild type and Gi+Gq animals (p = 0.94; n = 8).

(**D**) Quantification of NG2-positive pericytes along vessels in the GM at E17 after 0.75x CNO injection at E15 in wild type and Gi+Gq animals (p = 0.10; n = 7).

Disease Models & Mechanisms • Supplementary information

Table S1. Resources and reagents

REAGENT or RESOURCE	SOURCE	IDENTIFIER
**Antibodies**		
Rabbit anti-laminin	Sigma-Aldrich	Cat # L9393;<br>RRID: AB_477163
Biotinylated Isolectin B4	Vector Laboratories	Cat # B-1205;<br>RRID: AB_2314661
Rat anti-Ter119	BD Pharmingen	Cat # 553986;<br>RRID: AB_2650605
Rabbit anti-Phospho-p38	Cell Signaling	Cat # 4511;<br>RRID: AB_2139682
Rabbit anti-Collagen IV	Abcam<br>Bio-Rad	Cat# ab15633,<br>RRID: AB_302010<br>Cat # 2150-1470<br>RRID: AB_2082660
Rabbit anti-GFAP	Dako	Cat # Z 0334;<br>RRID: AB_10013382
Rabbit anti-Glut1	Thermo Scientific	Cat# RB-9052-P0;<br>RRID: AB_177894
FITC conjugated secondary antibodies	Jackson ImmunoResearch Laboratories	Cat # 111-095-003;<br>RRID: AB_2337972
Cy3 conjugated secondary antibodies	Jackson ImmunoResearch Laboratories	Cat # 711-165-152;<br>RRID: AB_2307443<br>Cat # 111-165-144;

Disease Models & Mechanisms • Supplementary information

		RRID: AB_2338006
FITC conjugated streptavidin	Jackson ImmunoResearch Laboratories	Cat # 016-010-084; RRID: AB_2337236
Peroxidase conjugated secondary antibodies	Santa Cruz Biotech	Cat # SC-2004; RRID: AB_631746 Cat # SC-2005; RRID: AB_631736
**Chemicals, Peptides, and Recombinant Proteins**		
S1P	Cayman Chemical	Cat # 62570
JTE-013	Cayman Chemical	Cat # 10009458
TY 52156	Tocris	Cat # 5328
CNO	APExBIO Tocris	Cat #: A3317 Cat #: 4936
**Critical Commercial Assays**		
Tyramide signal amplification (TSA) plus Cy3 kit	PerkinElmer, Waltham, MA	Cat # NEL744E001KT
GoTaq qPCR master mix	Promega	Cat # A6002 #TM318
**Experimental Models: Organisms/Strains**		

Disease Models & Mechanisms • Supplementary information

Ric8a conditional allele	Our own lab	Ma et al., 2012 *(40)*
S1pr1 conditional allele	JAX	JAX: 019141<br>Allende et al., 2003
Nestin-cre	JAX	JAX: 003771<br>Tronche et al., 1999
ROSA:LNL:tTA	JAX	JAX: 011008<br>Wang et al., 2008
TRE-hM3Dq	JAX	JAX: 014093<br>Alexander et al., 2009
TRE-hM4Di	JAX	JAX: 024114
**Oligonucleotides**		
GAPDH: F-tgcccccatgtttgtgatg, R- tgtggtcatgagcccttcc	Our own lab	
integrin β8: F-ccagtactgtgagaaggatgac, R-agttgacacagtgctgtgctga	Our own lab	
integrin β3: F-tggagacacctgtgtgagaag, R-ttcacctgctcgatgtcatc	Our own lab	
integrin β5: F-tcctgcttcgagagtgagtt, R-ttctccacagtgacactctcc	Our own lab	
integrin αv: F-gagtacttctcggtggtcct, R-ggggggctccaataaacaca	Our own lab	

Disease Models & Mechanisms • Supplementary information

Nestin: F-cgctcagatcctggaaggtg, R-ttctcagcctccagcagagt	Our own lab, this paper	
**Software and Algorithms**		
ImageJ		

Disease Models & Mechanisms • Supplementary information
